# Food addiction as a transdiagnostic feature associated with binge-eating symptoms in eating disorders: prevalence and rehabilitation outcomes in an Italian inpatient population

**DOI:** 10.1007/s40519-026-01861-5

**Published:** 2026-05-02

**Authors:** Margherita Boltri, Letizia Oprandi, Silvamaria Mastrocola, Giulia Cera, Federico Brusa, Carolina Gabutti, Valentina Villa, Francesca Manzo, Alberto Scalia, Emanuela Apicella, Sandra Savino, Gianluca Castelnuovo, Leonardo Mendolicchio

**Affiliations:** 1 Experimental Laboratory for Metabolic Neurosciences Research, I.R.C.C.S. Istituto Auxologico Italiano, 28824 Piancavallo, VCO Italy; 2 Psychology Department, Università Cattolica del Sacro Cuore, Milan, Italy; 3 Psychology Department, Università degli Studi di Torino, Turin, Italy; 4 Psychology Department, Università La Sapienza, Rome, Italy; 5 Laboratorio di Psicologia, I.R.C.C.S. Istituto Auxologico Italiano, Ospedale San Giuseppe, Piancavallo, VCO Italy; 6 U.O.C. dei Disturbi dell’Alimentazione e della Nutrizione, I.R.C.C.S. Istituto Auxologico Italiano, Ospedale San Giuseppe, Piancavallo, VCO Italy

**Keywords:** Food addiction, Eating disorders, Rehabilitation outcomes, Binge-eating symptoms

## Abstract

Food addiction (FA) is an emerging construct describing a compulsive and dysregulated pattern of food consumption that parallels mechanisms observed in substance-related addictions. This study aimed to investigate the prevalence and clinical correlates of FA in a sample of 141 Italian adult inpatients diagnosed with eating disorders (ED). Participants completed standardized self-report instruments assessing FA (YFAS 2.0), binge-eating behaviours (BES), ED-related psychopathology (EDI-3), body image distress (BUT), and general psychological symptoms (SCL-90) at admission (T0) and discharge (T1) from a multidisciplinary ED rehabilitation program. FA showed a high prevalence (85.6%), with more than half of the participants (51.1%) classified as severe. YFAS 2.0 scores were significantly higher among patients with binge eating disorder (BED) and bulimia nervosa (BN). FA severity was strongly associated with bulimic and binge-eating symptoms and interoceptive deficits, but not with body dissatisfaction. Regression analyses indicated that FA at admission was significantly associated with the severity of bulimic and binge-eating symptoms, independently of age, BMI, and illness duration. Overall, findings suggest that FA may reflect a dimensional feature associated with symptom severity across eating disorders, particularly in binge-spectrum presentations. Routine assessment of FA could therefore serve as a potentially useful clinical indicator and guide personalized interventions aimed at reducing compulsive eating behaviours and preventing relapse.

**Supplementary Information:**

The online version contains supplementary material available at 10.1007/s40519-026-01861-5.

## Introduction

In recent years, the concept of food addiction (FA) has attracted growing interest within the scientific community, raising significant questions regarding its definition, diagnostic validity, and clinical implications. Against the backdrop of rising global rates of obesity and eating disorders, it has been hypothesized that the consumption of energy-dense foods, particularly those high in sugar, fat, and salt, may elicit neurobiological responses comparable to those triggered by addictive substances [1, 2]. This hypothesis has led some researchers to propose the existence of a new form of addiction, namely *food addiction*. This proposed diagnostic entity is not yet officially recognized by major classification systems such as the DSM-5-TR [3]; however, it displays symptomatology that closely parallels that of substance use disorders. Food addiction is generally defined as a dysfunctional pattern of eating behaviour characterized by intense craving, loss of control, continued use despite negative consequences, tolerance, and withdrawal symptoms [4, 5]. Beyond behavioural patterns, neuroscientific evidence appears to support the hypothesis of a shared neurobiological foundation between food addiction and drug addiction. Specifically, the consumption of highly palatable foods has been shown to activate brain reward circuits, such as the mesolimbic dopaminergic system, similarly to the way addictive substances do [2, 6]. The most widely used tool to assess these traits is the Yale Food Addiction Scale (YFAS), now available in its updated version 2.0, which has been developed based on DSM-5 criteria for substance use disorders [7].

Epidemiological data indicate a variable prevalence of food addiction, ranging from 0 to 25% in the general population, with significantly higher rates observed in certain clinical subgroups such as eating disorders (ED), especially those with bulimia nervosa (BN) or binge-eating disorder (BED) [5]. One of the main controversies concerns the differentiation between food addiction and typical eating disorder-related features, commonly encountered in both BED and BN. While there are considerable symptom overlaps, some authors suggest that food addiction may represent a distinct condition, with a more pronounced neurobiological basis and a greater compulsive component compared to traditional eating disorders [5]. Others argue, however, that the concept of food addiction risks pathologizing common eating behaviours and that no “active substance” can be clearly identified in food, unlike drugs [1].

Specifically, BED and FA share several overlapping features, including the consumption of large quantities of food in the absence of hunger, food cravings, abdominal distension, and mood enhancement following food intake. In both cases, individuals often make unsuccessful attempts to eliminate the dysfunctional behaviour and experience negative psychological, physical, and social consequences, such as increased impulsivity and emotional instability [8, 9].

However, notable differences exist in the underlying function of the eating behaviour: individuals with FA tend to eat primarily in pursuit of pleasure, whereas those with BED are more likely to binge eat in response to psychological distress and concerns about weight, body image, or perceived personal failure [9, 10]. In addition, BED is typically marked by episodic behaviour, often occurring in isolation, and followed by feelings of guilt and shame. In contrast, the dysfunctional pattern in food addiction tends to be persistent and is generally unaffected by the presence of others. Another key distinction involves awareness of psychopathology: individuals with FA often deny or downplay the severity of their condition, whereas those with BED tend to demonstrate higher levels of insight [11]. It is important to emphasize that FA does not occur exclusively in conjunction with BED. According to recent scientific evidence, this phenomenon is also present in the general population and in individuals with other types of EDs. In particular, a significant co-occurrence of FA and BN has been observed [12]. Differentiating food addiction from other eating disorders and understanding its unique mechanisms is a critical goal for professionals working in the fields of eating disorders and mental health. Insight into the underlying psychopathological features can guide clinicians in developing more targeted and effective treatment strategies [9, 12]. Although eating disorders differ in their clinical presentation, increasing evidence supports a transdiagnostic perspective, emphasizing shared maintaining mechanisms such as impulsivity [13], emotion dysregulation [14], and altered reward processing [15]. From this perspective, FA may be particularly well-suited to a transdiagnostic conceptualization. Indeed, growing evidence suggests that FA symptoms are not confined to specific eating disorder diagnoses, but rather cut across diagnostic categories and are more closely associated with global psychopathological severity and dysregulated eating patterns. For instance, FA has been observed across anorexia nervosa, bulimia nervosa, binge-eating disorder, and obesity, with higher levels consistently linked to greater eating-related and general psychological impairment [12, 16]. Importantly, person-centred approaches further support this perspective. Using latent class analysis, Aloi et al. [17] identified distinct clinical profiles based on FA symptoms that were not strictly aligned with diagnostic categories but instead differed primarily in severity and associated psychopathology. Notably, individuals with and without binge-eating disorder were distributed across FA-based classes, suggesting that FA captures underlying dimensions of addictive-like eating that transcend categorical diagnoses. Overall, these findings support the conceptualization of FA as a transdiagnostic construct, reflecting shared mechanisms and severity gradients across eating disorders. This perspective provides a rationale for examining heterogeneous clinical samples as a unified group, while acknowledging that diagnosis-specific factors may still contribute to the observed associations. Thus, FA may represent a dimensional construct cutting across diagnostic categories, particularly in relation to loss-of-control eating behaviours. Adopting a transdiagnostic framework approaching FA across the ED spectrum might help highlighting similarities and differences across diagnostic subgroups.

The present study aimed to contribute to the growing body of research on this topic. Specifically, its primary objective was to estimate the prevalence rates and severity of FA in a sample of Italian inpatients with eating disorders. Secondary objectives included: (i) examining cross-sectional associations between FA and ED-related psychopathology, general psychological distress, and anthropometric variables; and (ii) exploring changes in FA and ED-related symptoms following a multidisciplinary inpatient rehabilitation program. An additional exploratory aim was to examine whether FA severity at admission was associated with the severity of binge-eating and bulimic symptoms at discharge. Given the exploratory nature of the study, the number of variables included in regression models was limited to a priori clinically relevant covariates (age, BMI change, and illness-related variables) in order to reduce the risk of overfitting.

## Methods

### Participants and procedure

**Fig. 1:**
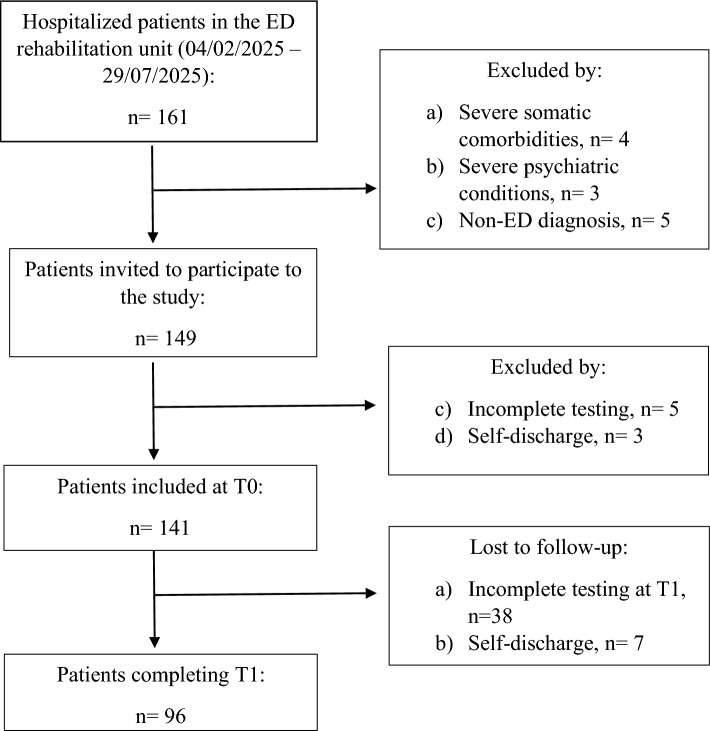
Patient eligibility flowchart (February–July 2025)

The study included 141 adults and young adults of both sexes, diagnosed with eating disorders (EDs) and admitted to the Eating and Nutrition Disorders Rehabilitation Unit at San Giuseppe Hospital in Piancavallo (VCO)–I.R.C.C.S. Istituto Auxologico Italiano. Inclusion criteria were: age between 18 and 66 years, diagnosis of an eating disorder according to DSM-5 or DSM-5-TR criteria [3, 18], and fluent knowledge of the Italian language. Exclusion criteria included the presence of non-ED-related illnesses (for example, neurodegenerative diseases) and severe psychiatric comorbidities. Prior to participation, all individuals provided written informed consent. Eligible participants completed self-administered questionnaires during the first week of admission (T0) and a few days before discharge (T1), taking into account that the standard rehabilitation program lasted 4 weeks; then based on the patient’s health condition, hospitalization could be extended by approximately 2 additional weeks. The study was conducted in accordance with the principles outlined in the Declaration of Helsinki and received approval from the local ethics committee (Comitato Etico Lombardia 5, Pr. no 351/25). Data collection took place between February and July 2025. A flow diagram describing participant recruitment, inclusion, and retention is provided in Fig. 1.

### Inpatient ED rehabilitation programme

Participants were admitted to a structured multidisciplinary inpatient rehabilitation programme specialized in ED treatment, with a primary focus on nutritional restoration and psychological stabilization. The standard care protocol included, when clinically indicated, enteral nutrition, supervised meals, individualized dietary planning with dietitian oversight, and nutritional psychoeducation groups. In addition, patients received physiotherapy and movement-based therapies (e.g. cycle ergometer, motor rehabilitation exercises, and postural gymnastics), psychiatric evaluations and consultations, individual psychological support, and group-based psychoeducational interventions. The overall treatment approach and core interventions were consistent across care tracks. However, patients presenting with severe malnutrition and admitted to the “acute track” were restricted from participating in group sessions and physiotherapy during the initial 2 weeks of hospitalization. The “acute track” had a total duration of approximately 6 weeks (2 weeks in acute care followed by 4 weeks in standard rehabilitation), whereas the standard track lasted around 4 weeks. Patients generally had freedom of movement within the unit and hospital facilities, except in cases where clinical conditions necessitated temporary restrictions. Treatment intensity was comparable across diagnostic groups; however, interventions were individualized according to specific clinical targets, such as weight restoration in restrictive profiles or reduction of binge/purge behaviours in binge-type profiles. For instance, psychotherapeutic group sessions were adapted based on patients’ symptomatic profiles: groups focused on binge eating or restrictive behaviours targeted symptom-specific strategies, while interventions addressing more transdiagnostic or social-emotional components, such as improving social engagement or interpersonal functioning, brought together patients across diagnostic categories. This approach reflects current clinical practice in specialized ED units, where both disorder-specific and transdiagnostic maintaining mechanisms are addressed. Although the program was not specifically designed to target food addiction, interventions addressing the binge-eating cycle and fostering patients’ psychological awareness of the circumstances, triggers, and mechanisms underlying their binge episodes may have contributed to a reduction in compulsive eating behaviours.

### Administration procedure

Assessments were conducted at two time points: T0, within the first 3 days of hospitalization, and T1, 1–2 days prior to discharge. Following discussion with the multidisciplinary team, eligible patients were invited to participate by research psychologists. Assessment sessions were scheduled within the patients’ daily therapeutic timetable, with small groups of 2–4 participants in a dedicated room within the unit. Administration conditions were standardized across both time points to ensure comparability. Each session lasted approximately 30 min and involved paper-and-pencil self-report questionnaires. The first section collected structured sociodemographic and clinical information, including illness duration, previous treatments, and comorbid conditions, followed by the Yale Food Addiction Scale 2.0 (YFAS 2.0) and additional self-report measures (see Psychometric Measures). At least one research psychologist was present during all sessions to provide clarification when necessary and to maintain standardized administration procedures. Anthropometric and clinical variables, including body weight and BMI, were obtained from patients’ medical records, as routine measurements were conducted by clinical staff but not disclosed to participants who requested not to receive this information. Attrition between T0 and T1 (*n = *45) was primarily due to logistical and organizational constraints within the inpatient unit. For example, self-discharge or early discharges (by 1 or 2 days) sometimes precluded scheduling follow-up assessments, and the availability of rooms and dedicated research personnel was limited to specific days of the week. As a result, some T1 assessments could not be completed. Retention at follow-up was therefore 68.1% (*n* = 96/141), which appears consistent with previously reported follow-up rates in inpatient eating disorder programs of similar duration [19].

### Measures

#### Demographic and anthropometric data

For each participant, descriptive data were collected, including gender, age, educational level, and previous diagnoses (if any). Patients also responded to self-administered questions regarding their eating disorder and its clinical course, including ED subtype, age at onset, waiting time before treatment initiation, and illness duration. Anthropometric data on body weight and body mass index (BMI) were recorded at T0 (at the beginning of the rehabilitation program) and T1 (at the end of treatment) in order to assess potential changes resulting from the inpatient rehabilitation process.

#### Psychometric measures

##### Binge-eating symptoms (BES)

The Binge Eating Scale (BES) is a 16-item self-report questionnaire designed to assess the severity of binge eating behaviours and the associated cognitive-affective symptoms in individuals with eating disorders [20]. Each item provides four response options, allowing participants to select the one that best reflects their personal experience. Higher scores indicate greater frequency and intensity of binge eating behaviours. Due to its good validity and ease of administration, the BES is currently one of the most widely used instruments in both clinical practice and scientific research [21].

##### Food addiction (YFAS 2.0)

The Yale Food Addiction Scale 2.0 (YFAS 2.0; [7]) was used to assess food addiction symptoms. The YFAS 2.0, developed to align with DSM-5 diagnostic criteria for substance use disorders, consists of 35 items assessing 11 symptoms typical of substance-related and addictive disorders. Items are rated on an 8-point scale ranging from “never” to “every day.” The total score ranges from 0 to 11, reflecting the severity of food addiction. The Italian version of the YFAS 2.0 has been validated in several studies, demonstrating strong psychometric properties and suitability for both epidemiological research and clinical use [22].

##### ED-related psychopathology (EDI-3)

The Italian version of the Eating Disorder Inventory-3 (EDI-3) [23, 24] was administered to assess psychopathological traits associated with EDs. This self-report questionnaire, originally developed by Garner and revised in 2004, comprises 91 items distributed across 12 primary scales: three ED-specific scales (drive for thinness, bulimia, and body dissatisfaction) and nine general psychological trait scales implicated in the development or maintenance of EDs (low self-esteem, personal alienation, interpersonal insecurity, interpersonal alienation, interoceptive deficits, emotional dysregulation, perfectionism, asceticism, and maturity fears). The EDI-3 also provides six composite scores (including “Eating Disorder Risk” and “General Psychological Maladjustment”). The 12-subscale structure and composite scores allow for an in-depth analysis of disorder-specific symptoms as well as broader maladaptive psychological traits, making the EDI-3 a versatile assessment instrument.

##### Body image distress (BUT)

Body image dissatisfaction and related subjective experiences were assessed using the Body Uneasiness Test (BUT; [25]), a self-report questionnaire composed of two sections. The first section includes 34 items rated on a 6-point Likert scale (0 = never, 5 = always) and evaluates five dimensions: weight phobia, concerns about body image, avoidance behaviours, compulsive self-monitoring, and feelings of detachment or depersonalization from the body. The second section comprises 37 items assessing concerns related to specific body parts or physiological functions. The BUT has recently been applied in longitudinal studies conducted in ED rehabilitation settings, demonstrating sensitivity to clinical changes over the course of intensive treatment [26].

##### Psychological distress (SCL-90)

Through the use of the Symptom Checklist-90 (SCL-90), developed by Derogatis and colleagues [27], it is possible to measure psychological symptoms experienced during the week preceding the assessment. This questionnaire consists of 90 items rated on a 5-point Likert scale (0 = not at all to 4 = extremely), allowing for the evaluation of both the severity and frequency of reported symptoms. The tool assesses 10 specific symptom dimensions (for example somatization, interpersonal sensitivity, and depression), as well as a Global Severity Index (GSI) that summarizes overall psychological distress. This structure enables a comprehensive and detailed overview of the main areas of psychopathological suffering and the specific symptom patterns characterizing the individual.

### Data analyses

All statistical analyses were conducted using Jamovi software (version 2.3.28). Descriptive statistics (means, standard deviations, and frequency distributions) were computed to summarize sociodemographic, clinical, and psychopathological characteristics of the sample across diagnostic groups. To examine differences in FA symptomatology across ED diagnoses, an analysis of covariance (ANCOVA) was performed with the Yale Food Addiction Scale 2.0 (Y-FAS 2.0) symptom count as the dependent variable, diagnosis as the between-subject factor, and body mass index (BMI), age, and illness duration as covariates. Post-hoc pairwise comparisons were conducted using Tukey correction for multiple testing. The distribution of FA severity levels (none, mild, moderate, and severe) across diagnostic categories was explored using descriptive analyses and graphical representations. Associations between FA symptoms and clinical variables were examined using Spearman’s rank-order correlations, given the non-normal distribution of several variables. Correlation analyses included measures of eating disorder psychopathology (EDI-3 subscales, BES), general psychopathology (SCL-90), and demographic and anthropometric variables. To examine the effect of FA severity on changes in eating disorder–related psychopathology over time, repeated-measures analyses of variance (RM-ANOVA) were performed, with FA severity as the between-subject factor and time (baseline vs. discharge) as the within-subject factor. Given the very small sample sizes of some diagnostic subgroups (AAN, *n *= 3; EDNOS, *n* = 10), main ANCOVA and RM-ANOVA analyses were conducted on the larger diagnostic groups (AN-R, AN-BP, BN, and BED) only. Finally, two multiple linear regression models were conducted to assess whether baseline FA severity was associated with bulimic symptoms (EDI-3 Bulimia subscale) and binge-eating symptoms (BES) at discharge, adjusting for potential confounders, including age, changes in BMI (ΔBMI z-score), and latency between illness onset and treatment seeking. Statistical significance was set at *p* < 0.05 for all analyses. Effect sizes (e.g. partial *η*^2^ and standardized beta coefficients) were reported where appropriate. The analyses were not pre-registered and should be considered exploratory. Although the main analytical approach was guided by a priori hypotheses and existing literature, no formal distinction between confirmatory and post-hoc analyses was predefined.

EDI-B and BES were selected as primary outcome measures for regression analyses due to their specific focus on bulimic and binge-eating symptomatology, which are theoretically and empirically most closely related to FA [28, 29]. While broader indices of ED psychopathology were also assessed, these measures were prioritized to test the hypothesis that FA is particularly associated with loss-of-control eating behaviours. Given the conceptual and item-level overlap between food addiction and binge-eating measures, FA was examined not as a fully independent construct but as a potentially overlapping dimension reflecting compulsive aspects of eating behaviour. Its inclusion was intended to evaluate whether it captures clinically relevant variance beyond traditional ED symptom measures [12].

## Results

### Sample characteristics

The total sample consisted of 141 participants, distributed across different diagnostic subgroups: atypical anorexia nervosa (AAN; *n* = 3), anorexia nervosa binge-purge subtype (AN-BP; *n* = 24), restrictive anorexia nervosa (AN-R; *n* = 45), binge eating disorder (BED; *n* = 36), bulimia nervosa (BN; *n* = 23) and eating disorders not otherwise specified (EDNOS; *n *= 10). With regard to gender distribution, the vast majority of participants were female (*n* = 133; 94.3%), whereas only a small proportion were males (*n* = 8; 5.7%). The mean age of the overall sample was approximately 32 years (SD = 13.6), with significant differences across diagnostic groups: BED patients were the oldest (M = 40 years, SD = 15.5), while those with AAN were the youngest (M = 20 years, SD = 1.73). Moreover, the average age of onset was situated in late adolescence (around 18 years), with earlier onset reported by individuals with AAN and BN (15 and 16 years, respectively).

The eating disorder had a mean duration of approximately 14 years (SD = 11.8), with considerable variation across subgroups: from 5 years in AAN to over 17 years in the EDNOS group. The mean interval between disorder onset and treatment seeking was relatively long (approximately 49 months), with particularly extended values in the BED, BN, and EDNOS groups, extending even beyond 55 months. Regarding educational level, the overall mean corresponded to just over 13 years of schooling (calculated from the beginning of primary education), with no substantial differences among groups. Therefore, many participants had obtained a high school diploma.

From an anthropometric perspective, body weight and body mass index (BMI) clearly reflected the clinical differences; indeed, patients with AN-R displayed the lowest values (mean BMI = 15.4 ± 4.39 kg/m^2^), whereas individuals with BED showed the highest (mean BMI = 38.2 ± 8.18 kg/m^2^). Self-report questionnaires highlighted differential patterns across groups. BED and BN patients obtained higher scores on the Binge Eating Scale (BES) and particularly elevated values on the Yale Food Addiction Scale (Y-FAS), indicating greater severity of food addiction-related symptoms. Data from the EDI-3 indicated comparable levels of drive for thinness across diagnostic categories, with the lowest mean score observed in BED patients; the bulimia subscale showed the highest scores in BN and BED, while body dissatisfaction was consistently elevated across all groups. Similarly, scores on the BUT (Body Uneasiness Test) did not reveal marked group differences, with overall moderate values.

Finally, general psychopathological distress assessed through the Symptom Checklist-90 (SCL-90) reported a mean score of 1.87 (SD = 0.79), with the highest scores recorded in the AN-BP and AN-R groups. Descriptive statistics grouped by diagnosis are reported in Table 1.

**Table 1:** Descriptive statistics of the sample (M ± SD) grouped by diagnosis at baseline

Variable	*N *= 141	AAN (*n* = 3)	AN-BP (*n* = 24)	AN-R (*n* = 45)	BED (*n *= 36)	BN (*n* = 23)	EDNOS (*n* = 10)	Min–max
Age	31.7 ± 13.6	20.0 ± 1.73	30.8 ± 11.0	25.8 ± 11.6	40.2 ± 15.5	30.1 ± 9.50	36.6 ± 14.9	18–66
Disease onset (age)	18.7 ± 9.83	15.0 ± 1.0	17.7 ± 8.58	17.8 ± 5.45	23.2 ± 15.6	15.7 ± 5.36	16.7 ± 4.85	0–56
Disease length (years)	13.6 ± 11.8	5.0 ± 1.0	15.7 ± 13.9	8.74 ± 10.8	16.8 ± 12.2	14.8 ± 8.42	17.7 ± 11.5	0–51
Latency onset- admittance to care (months)	48.9 ± 71.9	21.7 ± 15.6	53.0 ± 59.9	37.3 ± 92.6	58.8 ± 56.3	55.7 ± 69.0	57.4 ± 50.3	0–480
Educational level	13.2 ± 2.62	10.7 ± 2.52	13.3 ± 1.94	13.5 ± 3.04	12.9 ± 2.88	13.3 ± 2.12	13.8 ± 1.93	5–20
Weight (kg)	64.5 ± 32.8	63.3 ± 6.84	45.3 ± 6.53	38.2 ± 9.93	105 ± 30.3	69.4 ± 17.6	70.3 ± 21.3	14.1–236
BMI (kg/m^2^)	24.2 ± 10.9	23.4 ± 2.28	17.2 ± 2.01	15.4 ± 4.39	38.2 ± 8.18	25.8 ± 6.42	26.8 ± 7.69	10.3–67.3
BES	21.3 ± 10.4	17.3 ± 4.73	23.3 ± 11.0	14.8 ± 6.64	25.3 ± 10.3	29.0 ± 8.21	14.8 ± 8.61	1–44
Y-FAS 2.0 total	5.53 ± 3.46	3.67 ± 2.31	5.71 ± 3.26	3.32 ± 2.22	7.46 ± 3.50	7.91 ± 2.66	3.10 ± 2.77	0–11
EDI-3 DT	21.1 ± 7.23	21.0 ± 11.3	23.5 ± 6.87	21.9 ± 6.98	18.3 ± 7.29	21.9 ± 5.54	19.5 ± 9.43	0–28
EDI-3 B	10.9 ± 9.10	3.33 ± 4.93	13.2 ± 8.73	3.89 ± 4.90	15.2 ± 8.29	18.5 ± 8.04	7.10 ± 5.51	0–32
EDI-3 BD	29.2 ± 8.32	30.3 ± 11.6	30.9 ± 8.80	28.4 ± 8.64	29.2 ± 7.97	29.7 ± 6.72	27.4 ± 10.5	11–40
EDI-3 EDRC	61.2 ± 17.6	54.7 ± 25.3	67.5 ± 16.8	54.2 ± 16.2	62.6 ± 17.8	70.1 ± 12.1	54.0 ± 20.3	16–100
BUT PST	21.8 ± 9.55	23.0 ± 11.3	23.2 ± 9.92	22.9 ± 9.54	20.2 ± 9.83	21.9 ± 9.68	19.3 ± 8.12	3–37
BUT PSDI	3.06 ± 0.824	2.76 ± 0.332	3.15 ± 0.853	3.19 ± 0.897	2.92 ± 0.913	3.00 ± 0.592	2.92 ± 0.634	0–5
SCL-90 total	1.87 ± 0.794	1.71 ± 0.679	2.08 ± 0.780	2.03 ± 0.795	1.66 ± 0.934	1.84 ± 0.550	1.47 ± 0.607	0.08–3.60

*AAN* atypical anorexia nervosa, *AN-BP* anorexia nervosa binge-purge subtype, *AN-R* restrictive anorexia nervosa, *BED* binge eating disorder, *BN* bulimia nervosa, *EDNOS* eating disorders not otherwise specified, *BES* Binge Eating Scale, *YFAS 2.0* Yale Food Addiction Scale 2.0, *EDI-3* Eating Disorder Inventory-3rd edition (subscales: *DT* Drive for Thinness, *B* Bulimia, *BD* Body Dissatisfaction, *EDRC* Eating Disorder Risk Composite), *BUT* Body Uneasiness Test (*PST* Positive Symptom Total, *PSDI* Positive Symptom Distress Index), *SCL-90* Symptom Checklist-90

### Distribution of food addiction severity levels in the ED sample

**Fig. 2:**
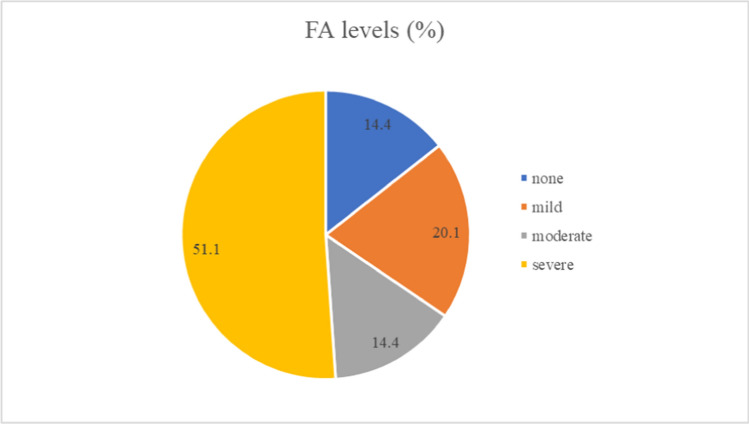
Overall distribution (%) of food addiction (FA) severity levels (none, mild, moderate, and severe) in the total sample

**Fig. 3:**
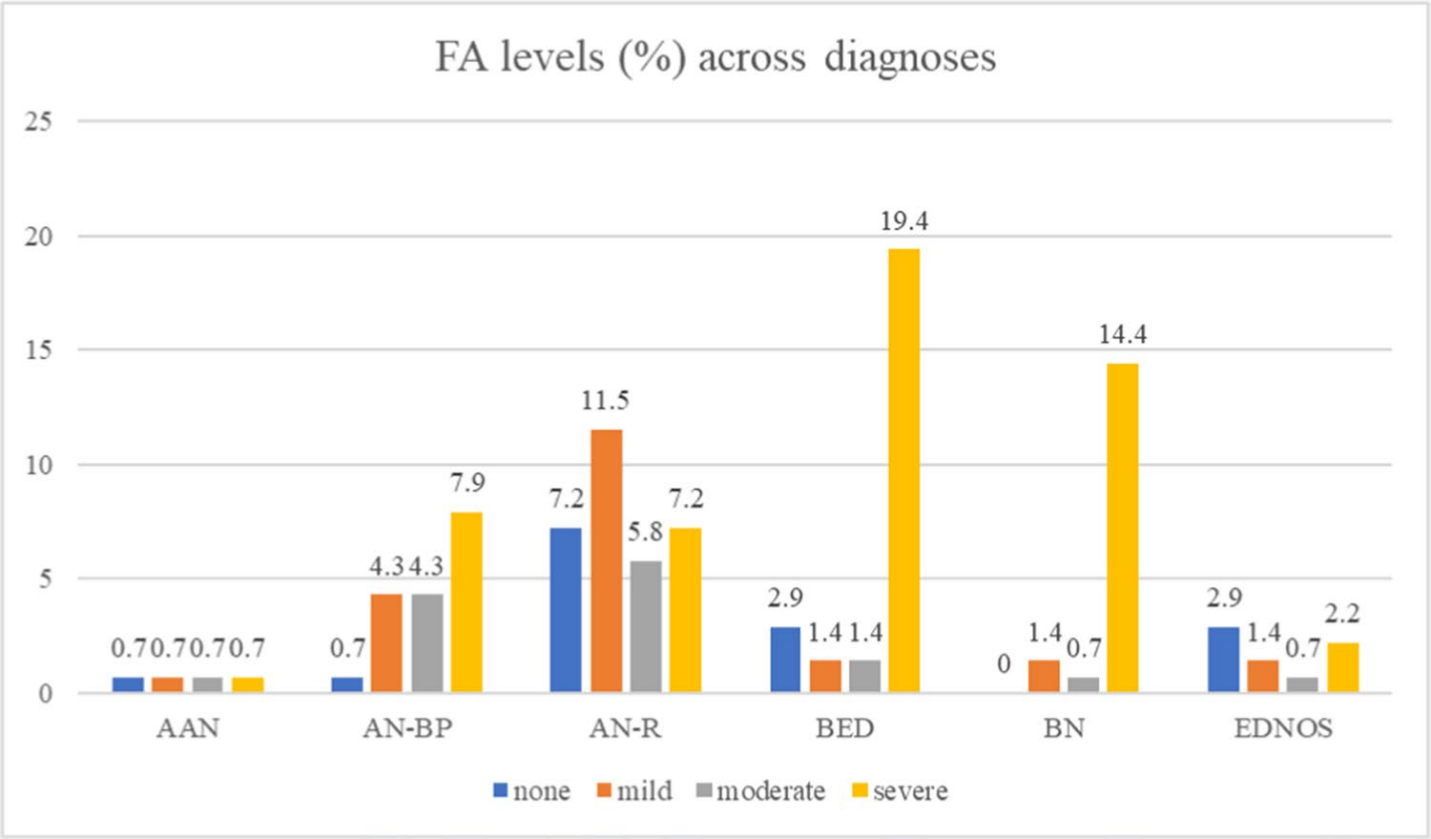
Distribution of FA severity levels (%) across eating disorder diagnoses: *AAN* Atypical Anorexia Nervosa, *AN-BP* Anorexia Nervosa–Binge/Purge subtype, *AN-R* Anorexia Nervosa–Restrictive subtype, *BED* Binge Eating Disorder, *BN* Bulimia Nervosa, and *EDNOS *Eating Disorder Not Otherwise Specified

One of the aims of our study was to investigate the prevalence rates of food addiction in patients diagnosed with eating disorders (EDs). To this end, the following figure illustrates the prevalence rates and the distribution of severity levels of food addiction (Y-FAS 2.0) across the different diagnostic groups. Specifically, it can be observed that patients with AN-R show a balanced presence of mild (*n* = 16; 11.5%), moderate (*n* = 8; 5.8%), and severe levels (*n* = 10; 7.2%), whereas in BED and BN individuals there is a clear predominance of the severe form (BED: *n* = 27; 19.4%–BN: *n* = 20; 14.4%). Conversely, in the AAN most cases fall within the moderate (AAN: *n* = 2; 1.4%) or absent categories (AAN: *n* = 1; 0.7%). In the EDNOS group, the distribution mainly concentrated in the severe (*n* = 3; 2.2%) and absent categories (*n* = 4; 2.9%). Moreover, in the AN-BP subgroup the distribution appears mixed, with mild (*n* = 6; 4.3%), moderate (*n* = 6; 4.3%), and especially severe cases (*n* = 11; 7.9%), although showing a stronger tendency toward the severe level. Overall, when collapsing across diagnoses, 20.1% (*n* = 28) of patients fell in the mild category, 14.4% (*n* = 20) in the moderate category, and more than half of the sample (51.1%; *n* = 71) in the severe category, while 14.4% (*n* = 20) did not meet criteria for food addiction. These results highlight the differential expression of food addiction symptomatology depending on diagnosis. Figures 2 and 3 display the distribution of FA severity levels (%) in the total sample and across diagnostic groups.

To further examine diagnostic differences in FA symptomatology, an ANCOVA was performed using the YFAS 2.0 symptom count as the dependent variable, diagnosis (AN-R, AN-BP, BN, BED) as the between-subject factor, and BMI, age, and illness duration as covariates. Small diagnostic subgroups (AAN, EDNOS) were excluded from these analyses to increase the reliability of between-group comparisons. The analysis revealed a significant main effect of diagnosis [F(3, 101) = 16.78; *p* < 0.001], while BMI was also a significant covariate [F(1,101) = 7.22; *p* = 0.008]. Post-hoc comparisons (Tukey corrected) indicated that patients with BED and BN exhibited significantly higher YFAS symptom scores compared to those with AN-R (both Ps < 0.001) and AN-BP (all Ps < 0.01). No significant differences were observed between BED and BN groups, both showing the highest FA symptom burden. These results confirm that the expression of food addiction symptoms varies across eating disorder diagnoses, with binge-type disorders showing the greatest overlap with addictive-like eating patterns. Full analysis is reported in Table S1 in Supplementary Materials.

### Associations between food addiction, demographic and clinical variables

Spearman’s correlations showed that YFAS 2.0 scores were strongly and positively associated with several indicators of eating disorder–related psychopathology (Table 2). Specifically, the Bulimia subscale (EDI-3 B) demonstrated a very high correlation with food addiction (*ρ* = 0.772, *p* < 0.001), as did the Binge Eating Scale (BES) (*ρ* = 0.741, *p* < 0.001), confirming the close relationship between loss-of-control eating behaviours and food addiction symptoms. Significant associations were also observed with the EDI-3 Eating Disorder Risk Composite (EDRC) (*ρ* = 0.435, *p* < 0.001) and Interoceptive Deficits (EDI-3 ID) (*ρ* = 0.218, *p* = 0.010), suggesting that greater levels of disordered eating and interoceptive dysfunction are linked to more severe food addiction features. Among general psychopathological measures, only the SCL-90 total score was significantly, albeit weakly, correlated with YFAS scores (*ρ* = 0.188, *p* = 0.029), whereas measures of body dissatisfaction and physical uneasiness (BUT_PTS and BUT_PSDI) were not. With regard to general psychopathology, YFAS 2.0 scores showed small to moderate correlations with several SCL-90 subscales, including Obsessive–Compulsive (*ρ* = 0.206, *p* = 0.016), Interpersonal Sensitivity (*ρ *= 0.208, *p* = 0.015), Hostility (*ρ* = 0.192, *p* = 0.025), Phobic Anxiety (*ρ* = 0.184, *p* = 0.032), Paranoid Ideation (*ρ* = 0.218, *p* = 0.011), and Psychoticism (*ρ* = 0.229, *p* = 0.007). These associations, although modest in magnitude, suggest that food addiction symptoms are linked to heightened obsessive–compulsive traits, interpersonal sensitivity, and subclinical psychotic-like or paranoid ideation. The complete correlation matrix between YFAS 2.0 and SCL-90 subscales is reported in Table S2 in Supplementary Materials. Regarding anthropometric and demographic variables, food addiction was positively associated with age (*ρ* = 0.229, *p* = 0.007), BMI (*ρ* = 0.301, *p* < 0.001), body weight (*ρ* = 0.330, *p* < 0.001), and the latency between disease onset and admittance to care (*ρ* = 0.257, *p* = 0.006). No significant correlations emerged with age at onset, illness duration, or educational level. Overall, these findings suggest that the severity of food addiction is more closely linked to binge eating symptoms and to the impulsive and interoceptive components of eating disorder psychopathology, as well as to higher anthropometric indicators.

**Table 2:** Spearman’s correlations between YFAS, demographic and clinical variables (T0)

Variable	*ρ* (Spearman’s rho)	*p*-value
ED-related psychopathology		
EDI-3 B	0.772***	< 0.001
EDI-3 DT	0.008	0.929
EDI-3 BD	0.050	0.558
EDI-3 EDRC	0.435***	< 0.001
EDI-3 ID	0.218**	0.010
BES	0.741***	< 0.001
Other psychopathological aspects		
SCL-90 total	0.188*	0.029
BUT_PTS	0.090	0.296
BUT_PSDI	0.056	0.518
Anthropometrics and demographics		
Age	0.229**	0.007
BMI (kg/m^2^)	0.301***	< 0.001
Weight (kg)	0.330***	< 0.001
Disease onset (age)	0.057	0.53
Disease length (years)	0.117	0.199
Latency onset–admittance to care (months)	0.257**	0.006
Educational level	0.060	0.484

*Y-FAS 2.0* Yale Food Addiction Scale 2.0, *EDI-3* Eating Disorder Inventory-3rd edition (subscales: *DT* Drive for Thinness, *B* Bulimia, *BD* Body Dissatisfaction, *EDRC* Eating Disorder Risk Composite, *ID* Interoceptive Deficits), *BUT* Body Uneasiness Test (*PST* Positive Symptom Total, *PSDI* Positive Symptom Distress Index), *SCL-90* Symptom Checklist-90

*p < 0.05, **p < 0.01, ***p < 0.001

### Effect of food addiction severity on ED-related psychopathology

**Fig. 4:**
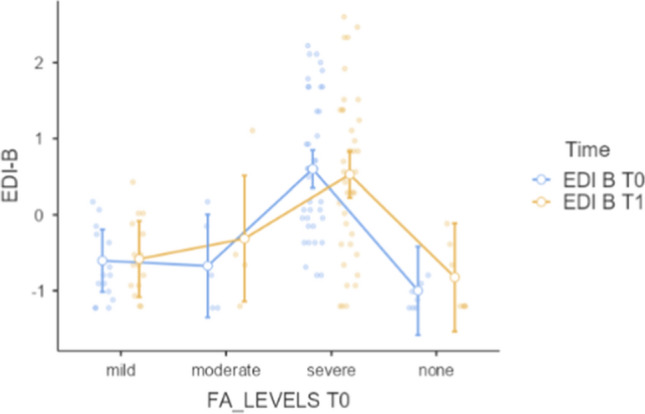
Observed and estimated mean EDI-B scores (± SE) across FA severity levels (mild, moderate, severe, none) at baseline (T0) and follow-up (T1). Individual data points represent participants’ scores

**Fig. 5:**
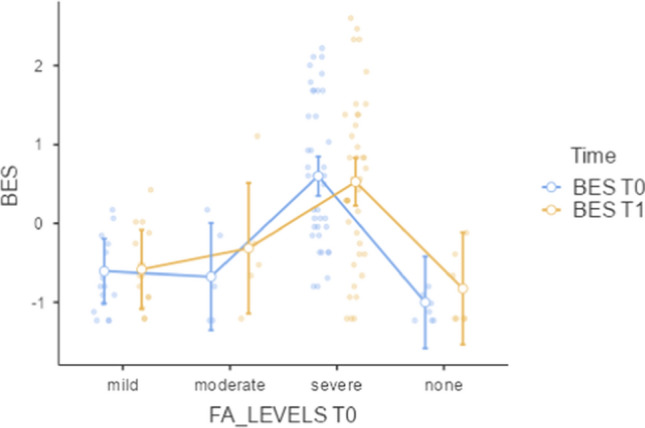
Observed and estimated mean BES scores (± SE) across FA severity levels (mild, moderate, severe, none) at baseline (T0) and follow-up (T1). Individual data points represent participants’ scores

At baseline (T0), participants with severe levels of food addiction (FA), as measured by the YFAS 2.0, exhibited significantly higher scores on both the Bulimia subscale of the EDI-3 (EDI-B) and the Binge Eating Scale (BES) compared to those with mild or no FA symptoms. Repeated Measures ANOVA confirmed a significant between-subjects effect of FA severity on EDI-B [F(3,59) = 12.69, *p* < 0.001] and BES [F(3,60) = 12.43, *p* < 0.001], with no significant time × FA interactions, indicating that these differences persisted at discharge (T1). Post-hoc comparisons revealed that participants with severe FA scored significantly higher than those with mild and no FA on both measures at baseline (EDI-B: vs. mild, *t *=  −4.677, *p* < 0.001; vs. none, *t* = 4.629, *p* < 0.001; BES: vs. mild, *t* =  −4.073, *p* < 0.001; vs. none, *t* = 5.321, *p* < 0.001). For EDI-B scores, the severe group also differed significantly from the moderate group (*t* =  −2.927, *p *= 0.029), whereas for BES scores, the difference between severe and moderate FA did not reach statistical significance (*t* = −1.872, *p* = 0.396). No significant differences were observed among mild, moderate, and no FA groups across both measures (all *p* > 0.05). No significant differences were found for other EDI-3 composite scores (e.g., EDI-3 EDRC) or general psychopathology (SCL-90), except for a modest association between FA severity and BMI change (ΔBMI z-score). Figures 4 and 5 display observed and estimated mean EDI-B and BES scores across FA severity levels at baseline and follow-up.

### Food addiction as a transdiagnostic feature associated with bulimic and binge-eating symptoms in EDs

Two multiple linear regression models were conducted to examine whether FA severity at admission (T0), was associated with bulimic and binge-eating symptomatology, controlling for changes in BMI, age, and latency between illness onset and admittance to care. FA severity modelled both as a continuous variable (Y-FAS FSC total score) and using a simplified categorical grouping (mild vs. severe) to provide complementary information. When modelling BES scores at discharge (T1) as the dependent variable, its association with FA severity resulted significant (*β* = 0.139, SE = 0.034, *t *= 4.07, *p* < 0.001, *β* standardized = 0.48, partial *η*^2^ = 0.21). Similarly, EDI-B scores at T1 were significantly associated with continuous FA severity (*β* = 0.180, SE = 0.033, *t *= 5.41, *p *< 0.001, *β* standardized = 0.61, partial *η*^2^ = 0.32), with none of the covariates showing significant effects in either model. Parallel analyses using simplified categorical FA levels indicated that participants classified as severe FA exhibited significantly higher EDI-B scores (*β* = 1.13, *p* < 0.001) and BES scores (*β* = 0.81, *p* = 0.005) compared to those with mild FA. No other variables, including ΔBMI (*p* = 0.63), age (*p* = 0.82), or latency onset (*p* = 0.69), resulted significant.

Taken together, these findings suggest that food addiction severity at admission may be a relevant factor associated with bulimic and binge-eating symptoms, independent of BMI changes, age, or illness duration, and that both continuous and categorical representations of FA severity provide complementary insights into its association with eating-disorder psychopathology. The results from models using FA as a continuous variable are reported in Tables 3 and 4, whereas the analyses using categorical FA levels are available in the Supplementary Materials (Table S3 and S4).

**Table 3:** Multiple regression models (BES T1)

Variable	*B*	SE	95% CI [Lower, Upper]	*β*	*t*	*p*	Partial *η*^2^
(Intercept)	0.11	0.11	[–0.11, 0.33]	–	0.99	0.327	0.21
Y-FAS FSC_T0	0.14	0.03	[0.07, 0.21]	0.48	4.07	< 0.001	–
ΔBMI (z-score)	–0.11	0.12	[–0.36, 0.14]	–0.10	–0.87	0.385	–
Age (z-score)	–0.11	0.12	[–0.36, 0.13]	–0.12	–0.91	0.364	–
Latency onset (z-score)	–0.03	0.11	[–0.25, 0.20]	–0.03	–0.25	0.805	–

Model summary: *F*(4, 62) = 9.17, *p* < 0.001, *R*^2^ = 0.37

**Table 4:** Multiple regression models (EDI-B T1)

Variable	*B*	SE	95% CI [Lower, Upper]	*β*	*t*	*p*	Partial *η*^2^
(Intercept)	0.068	0.108	[–0.15, 0.28]	–	0.63	0.530	0.32
Y-FAS FSC_T0 (continuous)	0.18	0.033	[0.11, 0.25]	0.61	5.41	< 0.001	–
ΔBMI (z-score)	0.028	0.12	[–0.21, 0.27]	0.03	0.23	0.818	–
Age (z-score)	–0.078	0.122	[–0.32, 0.17]	–0.082	–0.64	0.525	–
Latency onset (z-score)	–0.0007	0.109	[–0.22, 0.22]	–0.0008	–0.006	0.995	–

Model summary: *F*(4, 61) = 13.2, *p* < 0.001, *R*^2^ = 0.46

## Discussion

### Prevalence of food addiction in an Italian sample with eating disorders

In this study, the prevalence of food addiction (FA) among Italian patients with eating disorders (EDs) was 85.6% (51.1% severe), consistent with previous findings in similar clinical populations. Prior research has shown that FA is more frequent in individuals with EDs than in the general population [5]. A meta-analysis by Di Giacomo et al. [12] confirmed that, although binge-eating disorder is theoretically closest to the FA construct, its prevalence is comparable to or lower than that of Bulimia Nervosa, yet higher than in other ED subtypes. Studies have also reported notable rates of FA in patients with Anorexia Nervosa (AN). Cinelli et al. [30], for instance, found FA in 49.4% of Italian adolescents with EDs, including 53.7% in those with restrictive AN, indicating that FA may also be present in non–binge-type forms. Our results align with European multicentre studies [31, 32], which reported FA prevalence ranging from 35 to 97%, depending on diagnosis and assessment method. In a Spanish sample, FA was found in 54% of AN-R and 75% of AN-BP patients, with those affected showing more impulsive and dysregulated profiles [33]. However, the prevalence observed in the present study appears relatively high compared to previous reports. This may reflect the inpatient setting, characterized by greater illness severity and chronicity, as well as potential response bias. It is also possible that the YFAS 2.0 may over-identify FA symptoms in severely ill ED populations, raising questions about its specificity in this context.

Overall, international evidence indicates that FA prevalence is generally higher in binge-spectrum than in restrictive disorders [31, 34]. Our findings align with such evidence, and further support the view that FA may represent a transdiagnostic feature of EDs, reflecting compulsive and loss-of-control aspects of eating behaviour across diagnostic boundaries. However, given the higher representation and severity of binge-spectrum disorders (BN and BED), it is possible that the observed associations in our sample are partly driven by these subgroups rather than reflecting a fully transdiagnostic phenomenon.

### Food addiction as a transdiagnostic factor and severity indicator in eating disorders

*Food addiction* represents a transdiagnostic construct, present across various eating disorders and indicative of psychopathological severity. Multiple studies suggest that FA is more closely related to individual characteristics, such as age and difficulties in interoceptive processing, than to specific diagnostic categories [30]. Recent research has also documented an increase in compensatory behaviours and emotional eating during and after the COVID-19 pandemic, likely linked to prolonged stress, difficulties in emotional regulation, and disruption of daily routines [35, 36]. FA is frequently associated with mood disorders and personality disorders, particularly borderline personality disorder [37, 38], and shows significant correlations with BMI and self-reported depressive symptoms [39]. These findings indicate that FA severity reflects the interaction between psychological and physical factors in patients with eating disorders and may act as a maintenance factor, contributing to comorbid symptoms and representing a risk factor for relapse. Craving, loss of control, and dysregulation of the reward system can facilitate the persistence or return of dysfunctional symptoms even after targeted therapeutic interventions. Early identification and systematic assessment of FA in patients with eating disorders appears to be associated with bulimic and binge-eating symptoms, independently of BMI changes, age, or illness duration. Appropriate and adequate FA assessment can therefore lead clinicians to more personalized clinical interventions, aimed both at modulating reward mechanisms and preventing symptom relapse.

### Possible clinical phenotype

Our findings reveal a possible clinical phenotype delineated by a BMI in the normal-weight to overweight range accompanied by marked bulimic symptomatology, encompassing both compensatory behaviours and objective or subjective binge-eating episodes. Individuals within this subgroup tend to exhibit a more severe overall psychopathological burden, as reflected in their general psychopathological profiles. Notably, elevations emerge not in the anxiety or depressive domains but rather in subscales associated with cognitive disorganization, interpersonal dysfunction, and interoceptive impairment. Such dimensions are commonly linked to heightened distress, impulsivity dysregulation, and disturbances in self–other boundaries in eating disorders, aligning with previously proposed endophenotypic models [40]. Consistent with recent literature differentiating BED from FA [9], this phenotype does not exhibit marked body uneasiness. This pattern suggests that unlike individuals with BED, patients suffering from FA did not display heightened concern regarding body weight and shape, but rather rely on disordered eating behaviours as a psychological defence strategy. Furthermore, these patients tend to be older, display a later onset of the disorder, and show a longer latency between symptom emergence and access to treatment, suggesting a pattern consistent with greater chronicity. Overall, these findings raise the possibility that FA may function as a marker of symptom severity within binge-spectrum disorders, rather than a clearly separable diagnostic construct. Clarifying this distinction has important implications for both theoretical models and clinical practice.

### Implications for clinical practice

The regression model suggests that high levels of FA may hinder recovery in individuals with eating disorders, highlighting the need for systematic assessment of FA in clinical settings using validated measures. Identifying patients with elevated FA may allow clinicians to implement targeted interventions alongside standard treatments for eating disorders, potentially improving outcomes and promoting sustained recovery [41].

From a psychotherapeutic perspective, interventions addressing emotion regulation have gained increasing recognition in recent years [42, 43]. New approaches, such as Cognitive Remediation and Emotion Skills Training (CREST), provide structured programs aimed at enhancing emotion recognition and regulation, helping patients differentiate among emotions and understand their impact and implications [44]. Patients exhibiting addictive-like eating patterns may particularly benefit from these strategies, which also emphasize value-driven behaviours and the development of adaptive coping mechanisms to tolerate negative emotions without relying on food intake. Evidence from non-clinical adolescent populations further supports this approach, showing moderate positive correlations between difficulties in emotion regulation and binge eating, loss-of-control eating, and food addiction, suggesting that interventions targeting emotion regulation may help reduce risk behaviours and support recovery [45].

#### Speculative and emerging approaches targeting reward mechanisms involved in FA

In addition to psychological interventions, pharmacological strategies targeting the reward system may be beneficial. Glucagon-like peptide-1 receptor agonists (GLP-1RAs) have emerged as promising adjuncts, given their dual effects on appetite regulation and reward-related brain circuits. GLP-1 is an incretin hormone that modulates central dopamine pathways involved in reward processing, particularly within the ventral tegmental area, nucleus accumbens, and medial prefrontal cortex [46–48]. Clinical studies indicate that GLP-1RAs can reduce binge eating episodes, improve satiety, and support weight regulation without the psychiatric side effects associated with stimulant medications, making them a potentially safe and effective option for patients with BED or BN [49, 50]. While their application in anorexia nervosa remains under investigation, GLP-1 modulation may influence reward sensitivity and gut-brain signalling, warranting cautious exploration. Moreover, nutritional strategies may complement psychological and pharmacological interventions. Ketogenic diets (KDs), characterized by high fat and low carbohydrate intake, have shown promise in reducing cravings in both binge eating disorder and food addiction, likely by stabilizing brain metabolism and modulating reward pathways [51]. KD may offer a non-invasive adjunctive approach to manage compulsive food intake, particularly in patients presenting addictive-like eating behaviours, although psychological and body-image concerns still require concurrent therapeutic attention. Overall, these findings emphasize the importance of a multidisciplinary, personalized approach integrating emotion regulation training, reward-targeted pharmacotherapy, and nutritional interventions to optimize treatment outcomes for individuals with eating disorders and co-occurring FA. Overall, these approaches should be considered as avenues for future research rather than immediate clinical applications.

### Limitations and future directions

This study has several limitations. The sample was relatively small and characterized by a clear predominance of female participants. While this composition may limit the generalizability of the findings to other genders, it accurately reflects the actual clinical distribution of patients with eating disorders. This gender imbalance aligns with patterns commonly observed in clinical settings, where women are substantially more represented than men. Such disparity may partly stem from the persistent cultural perception of eating disorders as “female illnesses” and from men’s tendency to delay help-seeking behaviours [52]. Therefore, although the interpretation of results requires caution, the female predominance within the sample can nonetheless be viewed as an indicator of the ecological validity of the study, reflecting the clinical reality of eating disorder populations. Assessments relied exclusively on self-report measures and the rehabilitation period was short (4–6 weeks), with no long-term follow-up data. In addition, only Italian inpatients were included, and it would be valuable to examine the prevalence of food addiction (FA) in other clinical contexts, such as outpatient settings or patients in partial or full remission. To further assess the robustness of the findings, additional analyses yielded consistent patterns of association between FA and binge-eating measures, suggesting that the main effects are not solely attributable to small or less representative subgroups. Nevertheless, caution is warranted in interpreting FA as a fully transdiagnostic construct, and in generalizing these findings across all ED diagnoses. Further studies with larger and more balanced samples are needed to clarify the transdiagnostic validity of FA. In the original sample, some diagnostic subgroups were small (AAN *n* = 3,EDNOS *n* = 10), limiting the reliability of between-group comparisons. For this reason, these groups were excluded from the main ANOVA analyses, which were conducted on the larger and more homogeneous diagnostic groups (AN-R, AN-BP, BN, and BED). Within this restricted sample, binge-spectrum disorders (BN and BED) comprised the largest portions of the sample and exhibited the highest levels of food addiction and symptom severity. Therefore, it is possible that the observed associations primarily reflect patterns specific to these subgroups rather than a fully transdiagnostic phenomenon, suggesting the need for further research with larger and more balanced samples to confirm the transdiagnostic validity of FA. Future research should extend the investigation to additional diagnostic categories, such as ARFID, and consider the influence of comorbidities, which may act as confounding factors. Malnutrition may also impact cognitive functioning, potentially biasing questionnaire responses. Methodologically, increased use of the YFAS or the development of tools specifically designed to assess FA in eating disorder populations could improve measurement accuracy. The very high correlations observed between FA and binge-eating measures raise concerns regarding discriminant validity. This overlap suggests that FA may partly capture the same underlying construct as loss-of-control eating, rather than representing a fully distinct clinical entity. In addition, potential collinearity may have influenced regression results, and findings should therefore be interpreted with caution. Moreover, given the number of statistical tests performed, the risk of type I error cannot be excluded and findings should be considered exploratory and interpreted cautiously.

Finally, exploring correlations between FA and other functional markers, such as chronotype, could help clarify maladaptive behavioural cycles and support the development of targeted protocols to address FA and reduce relapse risk.

## Conclusion

Food addiction was highly prevalent in this inpatient ED sample and showed strong associations with binge-eating and bulimic symptoms, which is consistent with previous studies on the topic. However, given the cross-sectional nature of the main analyses and the absence of a control group, findings should be interpreted as associative rather than predictive. FA may represent a clinically relevant dimension linked to symptom severity, particularly within binge-spectrum disorders, although its incremental validity beyond established ED measures remains to be clarified. Future longitudinal and controlled studies are needed to better define its role in ED psychopathology and treatment.

## Supplementary Information

Supplementary material 1.

## Data Availability

The datasets generated and/or analyzed during the current study are not publicly available due to ethical and privacy restrictions related to sensitive clinical information. However, de-identified data that support the findings of this study are available from the corresponding author upon reasonable request.
