# Supplementary material for: Food addiction as a transdiagnostic feature associated with binge-eating symptoms in eating disorders: prevalence and rehabilitation outcomes in an Italian inpatient population
Source: Eat Weight Disord. 2026 May 2;31(1):62. doi: 10.1007/s40519-026-01861-5 (PMC13279732; doi:10.1007/s40519-026-01861-5)
Supplement: Supplementary file 1 — Supplementary material 1. [file 40519_2026_1861_MOESM1_ESM.docx]

**Supplementary Materials**

| **Table S1. ANCOVA of Y-FAS FSC scores by diagnosis, controlling for BMI, age, and illness duration**   \| Source \| SS \| df \| MS \| F \| *p* \| \| --- \| --- \| --- \| --- \| --- \| --- \| \| Diagnosis \| 402.77 \| 3 \| 134.26 \| 16.78 \| < .001 \| \| BMI_T0 \| 57.79 \| 1 \| 57.79 \| 7.22 \| .008 \| \| Age \| 3.55 \| 1 \| 3.55 \| 0.44 \| .507 \| \| Illness duration \| 20.31 \| 1 \| 20.31 \| 2.54 \| 0.114 \| \| Residuals \| 807.84 \| 101 \| 8.00 \| — \| — \|   *Note.* Y-FAS FSC_T0 = Food Addiction Scale total score at baseline. SS = sum of squares; df = degrees of freedom; MS = mean square; F = F statistic; p = significance value. |  |  |  |  |  |
| --- | --- | --- | --- | --- | --- | --- | --- | --- | --- | --- | --- | --- | --- | --- | --- | --- | --- | --- | --- | --- | --- | --- | --- | --- | --- | --- | --- | --- | --- | --- | --- | --- | --- | --- | --- | --- | --- | --- | --- | --- | --- |

**Post-hoc comparisons for diagnosis (Estimated Marginal Means)**

| **Comparison** | **Mean Difference** | **SE** | **df** | **t** | ***p* (Tukey)** | ***p* (Bonferroni)** | **Cohen’s *d*** |
| --- | --- | --- | --- | --- | --- | --- | --- |
| AN-BP vs AN-R | 3.15 | 0.79 | 101 | 4.01 | <.001 | <.001 | 1.113 |
| AN-BP vs BED | −4.03 | 1.40 | 101 | −2.88 | .0025 | .029 | −1.429 |
| AN-BP vs BN | −3.68 | 1.00 | 101 | −3.64 | .002 | .003 | −1.301 |
| AN-R vs BED | −7.18 | 1.43 | 101 | −5.02 | < .001 | < .001 | −2.542 |
| AN-R vs BN | −6.82 | 0.99 | 101 | −6.89 | < .001 | < .001 | −2.41 |
| BED vs BN | 0.36 | 1.09 | 101 | 0.33 | 0.988 | 1.00 | 0.128 |
| *Note.* Comparisons are based on estimated marginal means (EMMeans). SE = standard error; df = degrees of freedom; t = t statistic; p (Tukey) and p (Bonferroni) = significance values adjusted for multiple comparisons; Cohen’s d = effect size. | | | | | | | |

| **Table S2. Spearman’s correlations between Y-FAS 2.0 and SCL-90 subscales** | | | |
| --- | --- | --- | --- |
| **SCL-90 Subscale** | **ρ (Spearman)** | | ***p*** |
| Somatization | .134 | .119 | |
| Obsessive–Compulsive | .206 | .016* | |
| Interpersonal Sensitivity | .208 | .015* | |
| Depression | .123 | .153 | |
| Anxiety | .108 | .211 | |
| Hostility | .192 | .025* | |
| Phobic Anxiety | .184 | .032* | |
| Paranoid Ideation | .218 | .011* | |
| Psychoticism | .229 | .007** | |
| *Note.* *n* = 135. Spearman’s rank correlation coefficients between Y-FAS 2.0 total score and SCL-90 subscales. *p < .05, ** p < .01 | | | |

| **Table S3.** **Multiple Regression Model (EDI-B T1)** | | | | | | | |  |
| --- | --- | --- | --- | --- | --- | --- | --- | --- |
| Variable | B | SE | 95% CI (Lower–Upper) | β | *t* | *p* | Partial η² |  |
| FA level (severe vs. mild) | 1.13 | 0.29 | [0.56, 1.70] | 1.09 | 3.96 | < .001 | .29 |  |
| FA level (moderate vs. mild) | 0.29 | 0.42 | [–0.53, 1.12] | 0.28 | 0.71 | .48 | — |  |
| FA level (none vs. mild) | –0.23 | 0.36 | [–0.95, 0.49] | –0.22 | –0.64 | .52 | — |  |
| ΔBMI (z-score) | –0.06 | 0.12 | [–0.29, 0.18] | –0.05 | –0.48 | .63 | — |  |
| Age (z-score) | –0.03 | 0.12 | [–0.27, 0.21] | –0.03 | –0.24 | .82 | — |  |
| Latency (z-score) | –0.04 | 0.11 | [–0.25, 0.17] | –0.05 | –0.40 | .69 | — |  |
| Model summary: *F*(6, 68) = 5.46, *p* < .001, partial η² = .33 | | | | | | | | |

| **Table S4. Multiple Regression Model (BES T1)** | | | | | | | |
| --- | --- | --- | --- | --- | --- | --- | --- |
| Variable | B | SE | 95% CI [Lower, Upper] | β | *t* | *p* | Partial η² |
| (Intercept) | –0.14 | 0.13 | [–0.39, 0.12] | — | –1.05 | .299 | — |
| FA level (moderate vs. mild) | 0.27 | 0.42 | [–0.56, 1.10] | 0.26 | 0.66 | .514 | — |
| FA level (severe vs. mild) | 0.81 | 0.28 | [0.25, 1.37] | 0.79 | 2.89 | .005 | .21 |
| FA level (none vs. mild) | –0.44 | 0.36 | [–1.16, 0.28] | –0.43 | –1.23 | .224 | — |
| ΔBMI (z-score) | –0.19 | 0.12 | [–0.42, 0.05] | –0.17 | –1.59 | .117 | — |
| Age (z-score) | –0.06 | 0.12 | [–0.29, 0.17] | –0.06 | –0.50 | .621 | — |
| Latency onset (z-score) | –0.08 | 0.11 | [–0.29, 0.13] | –0.09 | –0.77 | .445 | — |
| Model summary: *F*(6, 69) = 4.69, *p* < .001, partial η² = .29 | | | | | | | |
